# Interactions between extracellular vesicles and microbiome in human diseases: New therapeutic opportunities

**DOI:** 10.1002/imt2.86

**Published:** 2023-02-06

**Authors:** Rongjin Luo, Yanmin Chang, Huaizhen Liang, Weifeng Zhang, Yu Song, Gaocai Li, Cao Yang

**Affiliations:** ^1^ Department of Orthopaedics, Union Hospital, Tongji Medical College Huazhong University of Science and Technology Wuhan China; ^2^ Department of Spine Surgery, Honghui Hospital Xi'an Jiaotong University Xi'an China; ^3^ Department of Neurology, Union Hospital, Tongji Medical College Huazhong University of Science and Technology Wuhan China

**Keywords:** biotherapy, extracellular vesicles, microbiome, microbiome–host interaction, vaccine

## Abstract

In recent decades, accumulating research on the interactions between microbiome homeostasis and host health has broadened new frontiers in delineating the molecular mechanisms of disease pathogenesis and developing novel therapeutic strategies. By transporting proteins, nucleic acids, lipids, and metabolites in their versatile bioactive molecules, extracellular vesicles (EVs), natural bioactive cell‐secreted nanoparticles, may be key mediators of microbiota–host communications. In addition to their positive and negative roles in diverse physiological and pathological processes, there is considerable evidence to implicate EVs secreted by bacteria (bacterial EVs [BEVs]) in the onset and progression of various diseases, including gastrointestinal, respiratory, dermatological, neurological, and musculoskeletal diseases, as well as in cancer. Moreover, an increasing number of studies have explored BEV‐based platforms to design novel biomedical diagnostic and therapeutic strategies. Hence, in this review, we highlight the recent advances in BEV biogenesis, composition, biofunctions, and their potential involvement in disease pathologies. Furthermore, we introduce the current and emerging clinical applications of BEVs in diagnostic analytics, vaccine design, and novel therapeutic development.

## Highlights

- Bacterial extracellular vesicles (BEVs) are key intermediators of microbiota–host communications by transporting their versatile bioactive cargoes, including proteins, nucleic acids, lipids, and metabolites.
- BEVs are proposed to exert both positive and negative roles in diverse physiological and pathological processes.
- BEVs secreted by pathogenic bacteria are strongly associated with the onset and progression of various human pathologies.
- BEV‐based platforms show promising clinical potential, including application in diagnostic analytics, vaccine design, and novel therapeutic development.

## Graphical abstract

Bacterial extracellular vesicles (BEVs) derived from various commensal and pathogenic bacteria have been shown to participate in a wide range of physiological and pathological processes by transporting versatile bioactive molecules, including proteins, nucleic acids, lipids, and metabolites. BEVs are intensively involved in the onset and progression of various diseases and BEV‐based platforms possess great potential in diagnostic analytics, vaccine design, and novel therapeutic development.

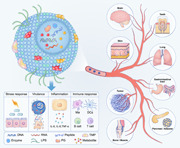

## INTRODUCTION

It is well established that the human body is a symbiotic ecosystem that contains a diverse range of commensal microorganisms, including eukarya, bacteria, and archaea, collectively termed microbiota [1, 2]. More than 100 trillion microbial cells, outnumbering human cells by 10 to 1, are estimated to inhabit almost every niche of the human body, especially the skin, oral cavity, respiratory, gastrointestinal, and urogenital tracts [3, 4]. Metagenomic sequencing data indicate that the human microbiome contains approximately 8 million unique protein‐coding genes, which is 360 times higher than the total number of human genes (between 20,000 and 25,000) [5, 6]. The composition and quantity of the microbiota community are evolutionarily maintained in homeostasis and perform specific genetic and metabolic functions in human health. However, dysbiosis of the microbiome, caused by internal and external factors in certain circumstances, may result in the onset of various diseases, including inflammatory bowels, autoimmune, metabolic, psychological/neurological diseases, and cancer [7, 8, 9, 10]. Moreover, with our rapidly advancing understanding of microbiota–host interactions and their regulatory networks, it is becoming increasingly clear that the microbiome is of great importance for the analysis of the host disease state and the development of novel therapeutic strategies.

Extracellular vesicles (EVs) are nano‐sized, heterogeneous membrane‐bound structures that are secreted by nearly all types of cells. They carry a diverse array of biologically active molecular cargos from parent cells, including lipids, proteins, nucleic acids, and small molecules, and serve as key intermediaries of intercellular communications [11, 12, 13]. Accumulating evidence has demonstrated that EVs are responsible for the complex and bidirectional relationship between microbiota and host cells, exerting an essential role in microbiota–host communications that are heavily involved in the regulation of various pathophysiological processes such as inflammation, angiogenesis, nutrient metabolism, and adaptive and innate immunity responses [14, 15, 16, 17]. Accordingly, diverse diagnostic and therapeutic approaches have been designed and clinically applied based on our increased understanding of the pivotal role of EVs in regulating microbiota–host connections [18, 19, 20, 21].

**Figure 1:**
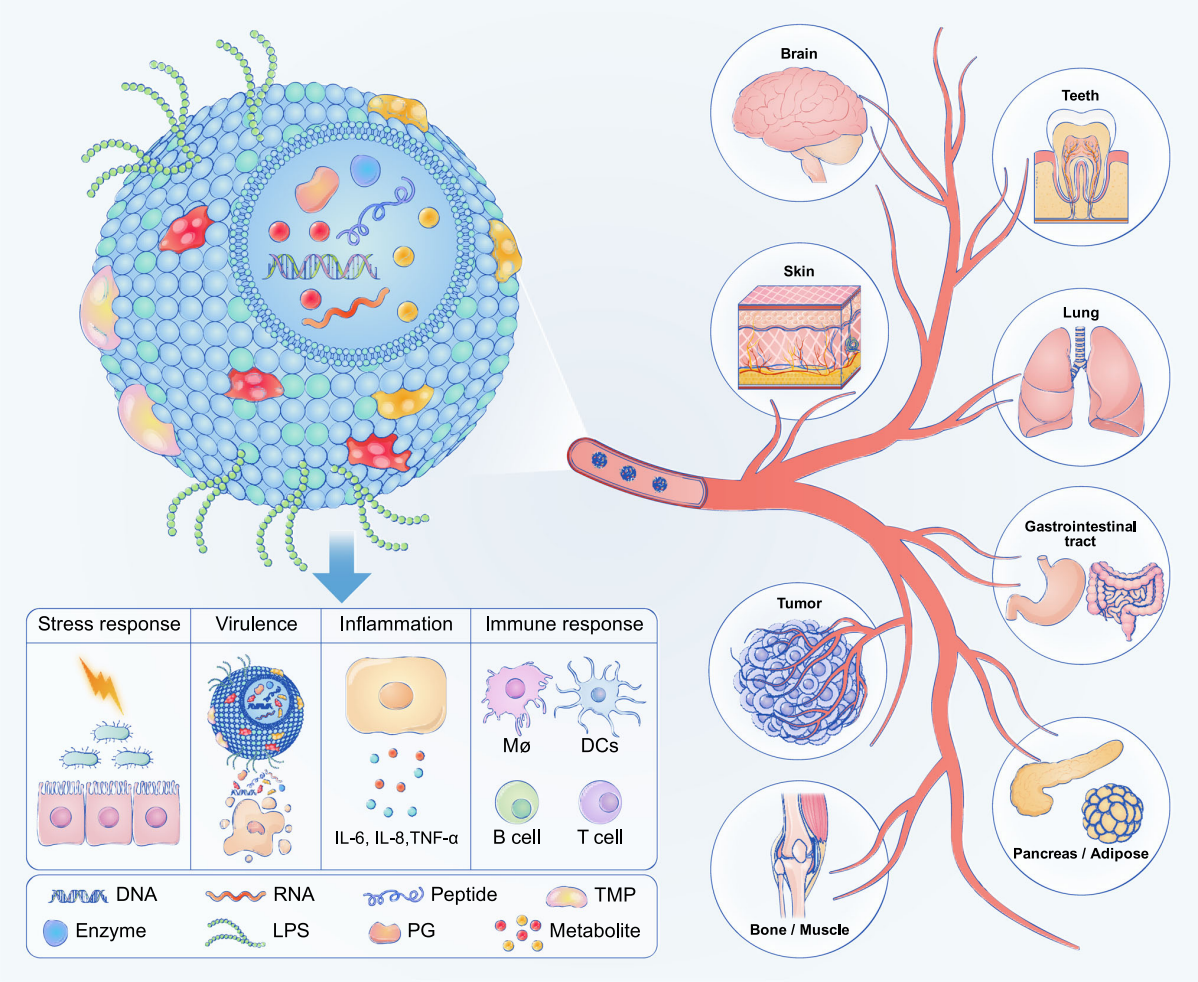
Schematic illustration of bacterial extracellular vesicles composition, biological functions, and their clinical correlations with various organs and tissues. DC, dendritic cell; IL‐interlekin; LPS, lipopolysaccharide; PG, peptidoglycan; TMP, transmembrane prtein; TNF, tumor necrosis factor.

Recently, EV‐mediated interactions between the microbiome and the host have attracted much attention and have been intensively investigated. The biological properties of eukaryotic EVs have been well described. In this review, we mainly highlight the current state of knowledge of microbiota‐secreted EVs, especially bacterial EVs (BEVs), focusing on their origin, biogenesis, composition, and, more specifically, their role in pathogenesis. Finally, we discuss the advances in BEV‐based clinical utilization, including diagnostics, vaccines, and novel therapeutics (Figure 1).

## ORIGIN, CLASSIFICATION, AND BIOGENESIS OF BEVs

Nowadays, the process of EV production has been clearly identified in nearly all cell types, including eukaryotic and prokaryotic cells. As early as 1967, researchers observed with electron microscopy, a process of spherical membrane‐bound structures being released in the form of bulging out and pinching off from part of the cell wall of *Vibrio cholerae* (Gram‐negative bacteria), and it was postulated to be relevant to the excretion product with cholera toxin [22]. Subsequently, in the early 1980s, EVs were found to be secreted from maturing sheep reticulocytes and function as a mechanism for iron transfer [23]. In 2007, the identification of abundant functional RNA present in EVs suggested a crucial role for EVs in cell‐to‐cell communication by RNA delivery [24]. Over the past two decades, several researchers have elucidated the characteristics of EV biogenesis, composition, biofunction, and clinical applications, especially EVs derived from eukaryotes. Researchers have reached a consensus that EV production is a highly regulated process, transporting diverse bioactive molecules into recipient cells either in their microenvironment or in distant tissues, initiating a variety of essential cellular signal transduction events [11, 25, 26].

As mentioned above, EVs are the generic name for cell‐derived membrane vesicles that vary in origin, size, and composition. To date, diverse classification methods have been introduced to define and distinguish each subset of EVs, but an accurate and standard classification method is still lacking [27, 28, 29]. Currently, eukaryotic EVs are generally categorized into three main subgroups depending on their biogenesis, size, and secretion: exosomes (30–150 nm), ectosomes or microvesicles (100–1000 nm), and apoptotic bodies (1000–10,000 nm) [29, 30]. Among the subtypes of EVs, exosomes are the most intensively studied nanovesicles. They are formed by inward invagination of the endosomal membrane, resulting in the formation of intraluminal vesicles (ILVs) within multivesicular bodies (MVBs). MVBs can either be directed to degradation by fusion with lysosomes, or directed to release their intraluminal vesicle cargo as exosomes. In contrast to exosomes, ectosomes are larger nanovesicles released directly from plasma membrane budding. Apoptotic bodies are large EVs released from apoptotic cells and eventually eliminated via phagocytosis [25, 31]. Eukaryotic EV generation and secretion is a complicated and finely tuned process and has been thoroughly summarized in the literature [25, 31, 32, 33, 34, 35]. Moreover, EVs are highly heterogeneous in nature, and the biophysical and biochemical properties of EVs often overlap between EV subtypes. It is important to understand the EV subtypes and their individual features before exploring their functional properties.

**Figure 2:**
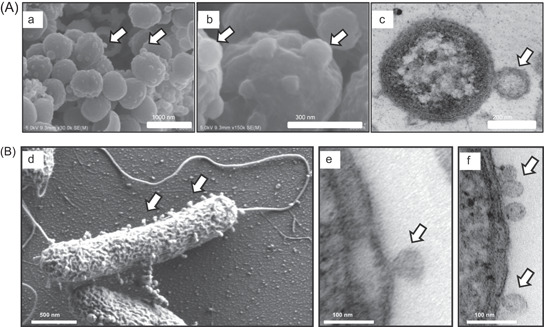
Visualization of BEVs release. (A) Cultured *Porphyromonas gingivalis* was observed with SEM (a, b) and TEM (c), representative graphs of whole cells with attached BEVs were illustrated. Reproduced with permission [37]. Copyright 2020, Elsevier. (B) BEVs detachment from *Vibrio harveyi* was observed with TEM (d) and SEM (e, f). Reproduced with permission [38]. Copyright 2018, American Society for Microbiology. The arrows indicate BEVs. BEV, bacterial extracellular vesicle; SEM, scanning electron microscope; TEM, transmission electron microscopy.

Since the importance of microbiota for human health has been revealed over the past decades, microbiota‐derived vesicles (MEVs) are an emerging topic of study. The human body harbors a variety of microorganisms including yeasts, archaea, parasites, viruses, and protozoa, of which bacterial taxa are currently the most intensively studied [36]. BEVs are nanovesicles naturally produced by all bacteria as part of their normal growth, ranging from approximately 20 to 300 nm. Specifically, BEVs released by Gram‐negative bacteria and Gram‐positive bacteria are referred to as outer membranes vesicles (OMVs) and membrane vesicles (MVs), respectively [14, 18] (Figure 2A,B). The mechanisms of BEV biogenesis in Gram‐negative and Gram‐positive bacteria follow two very different patterns based on their morphology, structure, and composition. OMVs were first found to originate from controlled blebbing of the outer membrane, which contains periplasmic contents, including outer membrane proteins, lipoproteins, and lipids [39, 40]. In addition, recent studies have revealed another type of OMV, called outer‐inner membrane vesicles (OIMVs), which are formed by fission of a protrusion of the outer and plasma membranes or explosive cell lysis and entrap both periplasmic and cytoplasmic components, including DNA and ATP [41, 42, 43, 44]. In contrast to Gram‐negative bacteria, Gram‐positive bacteria lack outer membranes but possess an extremely thick peptidoglycan (PG) cell wall, which is thought to prevent vesicular secretion. Emerging research has confirmed the existence of MVs derived from abundant Gram‐positive bacteria, which are postulated to originate from the blebbing of membrane material and encapsulate diverse membrane and cytoplasmic components [39, 45, 46, 47]. In addition, several membranous structures, referred to as nanotubes, nanowires, or nanopods protruding from the cytoplasmic membrane of Gram‐positive bacteria or the outer membrane of Gram‐negative bacteria, are also considered simple versions of BEVs [48, 49, 50]. Despite the different patterns of BEV production, the secretion of BEVs is an evolutionarily conserved characteristic among microbiota, suggesting the critical role of BEVs in the physical growth and survival of microbiota.

**Figure 3:**
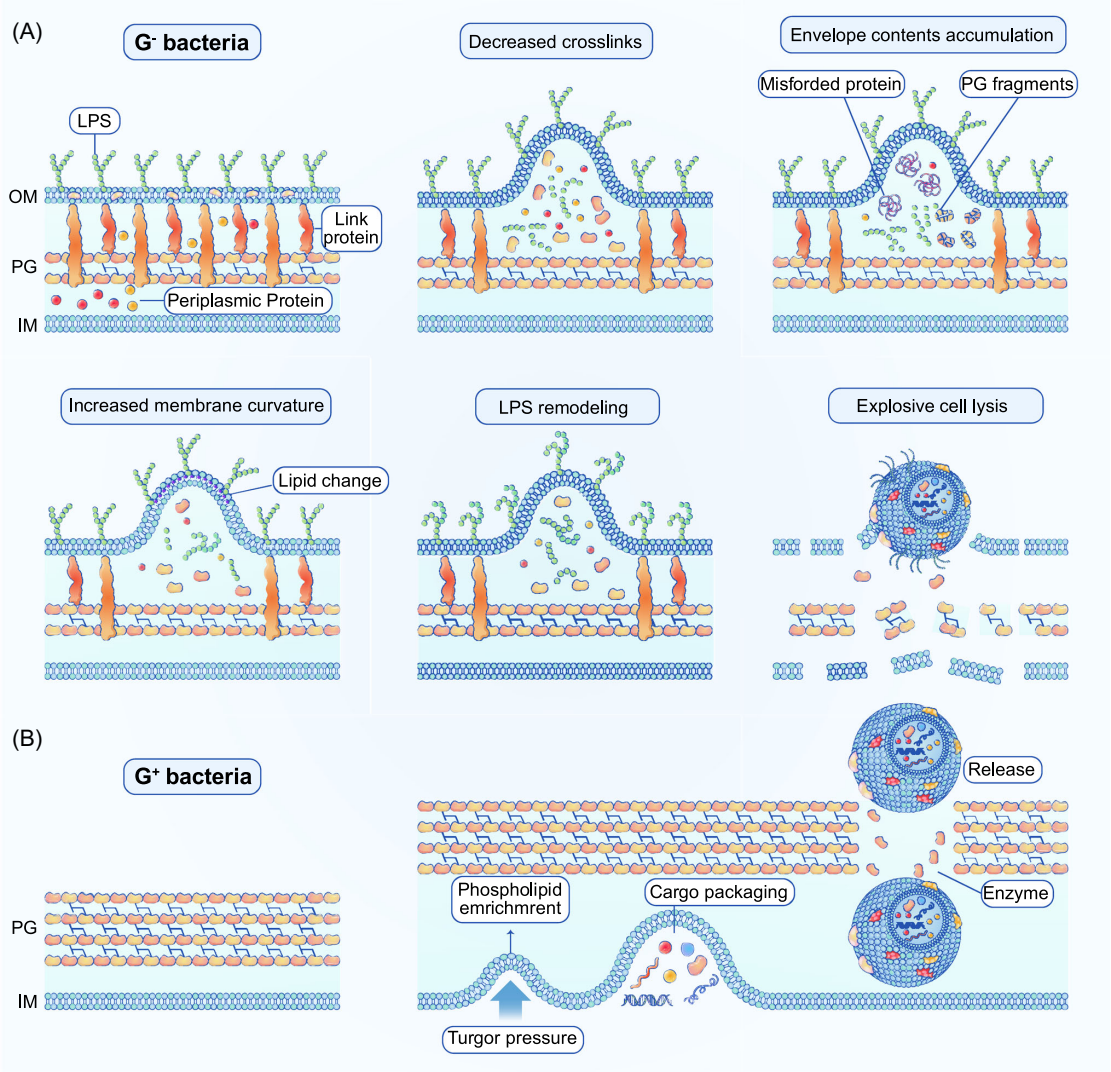
Potential mechanisms of BEVs biogenesis. (A) Gram‐negative bacteria cell envelope structure and related BEVs generation models, including decreased cross‐links between PG layer and outer membrane, local pressure overload, increased outer membrane curvature, LPS remodeling, and explosive cell lysis. (B) Gram‐positive bacteria cell envelope structure and related BEVs generation machinery. BEV, bacterial extracellular vesicle; LPS, lipopolysaccharide; PG, peptidoglycan.

EV generation in eukaryotes is mainly mediated by endosomal sorting complexes required for transport pathway proteins and their homologues, which have been well documented in many excellent studies. However, the mechanisms of BEV biogenesis in bacteria remain poorly understood. To date, a series of models for OMV formation in Gram‐negative bacteria have been proposed, based on genetic and biomedical studies (Figure 3A). The first model is a decreased interaction between the outer membrane and the PG layer, and PG‐associated lipoproteins, such as Lpp and the Tol‐Pal system, are a major class of surface proteins that are covalently anchored to the outer membrane and maintain membrane integrity and permeability. Lpp and Tol‐Pal mutations or their absence have previously been found to be associated with the increased release of periplasmic proteins and the formation of OMVs [51–53]. In the second model, local turgor pressure overload and imbalanced accumulation of multiple molecules, such as PG fragments, lipopolysaccharide (LPS), and misfolded proteins, into a defined area of the bacterial periplasm results in a strong pressure in the periplasmic space, deforming the upper outer membrane, leading to vesicle release [54, 55]. In the third model, changes in membrane physicochemical properties and asymmetric distribution of phospholipids between the inner and outer leaflets of the outer membrane are proposed to initiate membrane curvature, leading to asymmetric membrane expansion, bulging, and subsequent release of OMVs [56, 57]. In the fourth model, LPS remodeling is linked to OMV production, which yields two different types of LPS: the A‐band LPS (neutral charge) and the B‐band LPS (anionic charge). Only B‐band LPS was found packaged into OMVs, and mutants that only produce B‐band LPS were detected with increased OMV production [58, 59]. Moreover, in *Porphyromonas gingivalis*, conformational alterations of LPS increase its membrane curvature and consequently augment OMV production [60, 61]. Explosive cell lysis was reported to be an active OMV secretion system, mediated by cryptic prophage endolysin, a key enzyme required for cytoplasmic content release; endolysin‐deficient mutants are defective in MV production [44]. By contrast, Gram‐positive bacteria possess thick PG cell walls compared with Gram‐negative bacteria, suggesting the existence of a distinctly different mechanism of MV production. One hypothesis is that the accumulation of phospholipids in the outer leaflet of the membrane leads to rapid extension and outward bulging of the cytoplasmic membrane; turgor pressure elicited by the hypotonic environment may further promote the formation of MVs [62–65]. Mass spectrometry proteomic analyses of MVs revealed the presence of multiple PG‐degrading enzymes, such as transpeptidases and autolysins, inside the MVs, suggesting that cell wall modification or loosening by enzymes may enable the trafficking of MVs [66–69] (Figure 3B). Moreover, protein channels or structural cables in the PG cell walls are postulated to be another mechanism of MV release [45]. Hence, research on MV biogenesis in Gram‐positive bacteria is still in its infancy, and diverse internal and external factors affecting membrane fluidity and cell wall integrity appear to be critical determinants of MV generation.

BEV production is a well‐orchestrated and complex process that involves multiple regulatory factors. Genetic regulation of bacterial vesiculation has been widely introduced in a variety of studies, of which a genetic network composed of nearly 150 genes has been implicated in the regulation of vesiculation; loss of function mutation of specific genes could result in significant changes in the quantity and cargo content of BEV production [56, 70–72]. Furthermore, environmental stress factors, including exposure to antibiotics or unfavorable bacterial growth conditions, such as aberrant temperature or pH, have also been reported to modulate BEV production [73–76]. Nutrient availability and growth phages from which BEVs are isolated have been shown to influence BEV secretion and cargo packaging [77–79]. Hence, these observations led us to speculate that microbiota can dynamically optimize BEV production and selectively sort cargoes into BEVs based on their own circumstances.

## CARGO PACKAGING INTO BEVs

Generally, BEVs incorporate different biological components, including proteins, lipids, and nucleic acids, according to their parental cells. The quantity and variety of cargo selectively packaged into BEVs are influenced by their producer bacteria and environmental factors, which largely determine their distinct biofunctions under certain conditions. Thus, researchers have introduced various approaches and techniques, such as next‐generation sequencing (NGS), electron microscopy, mass spectroscopy, and proteomic analysis, to decipher the structure and composition of BEVs and subsequently investigate their role in diverse pathophysiological processes. However, it is still a great challenge to thoroughly interpret the composition of BEVs and the specific mechanisms by which cargo is selectively enriched in BEVs. In this section, we briefly recapitulate the general information on BEV composition and associated mechanisms.

***Proteins***. The protein content of BEVs has been validated in nearly all strains of bacteria, which are mainly composed of outer membranes, periplasmic, and cytoplasmic proteins [69]. With the rapid advancement of proteomic techniques, more than 3000 BEV‐associated proteins have been identified, which can be functionally classified as structural proteins, porins, ion channels, transporters, enzymes, and proteins related to stress responses [80–83]. Global proteomics highlights the unique protein profiles of BEVs, which endow them with distinct functional capabilities, such as signal transduction, pathogenicity, biofilm formation, metabolism, antibiotic resistance, and bacterial survival [38, 84–87]. The distinct features of dynamic changes in protein composition and BEV yield efficiency when bacteria are grown under different environmental conditions, suggests that cargos in BEVs may be selectively enriched compared to their parent bacteria, although few studies have determined the specific sorting mechanisms. For instance, the protein composition of BEVs released by *Mycobacterium avium* cultured in a metal mix medium mimicking the phagosome environment is distinct from that of BEVs produced in minimal medium [88].

***Lipids***. Lipids are the main structural constituents of BEVs' biological membranes and are mostly derived from the cytoplasmic membrane and endomembrane. Consistently, experimental evidence from *Escherichia coli* revealed a conserved lipid composition between the outer membranes and BEVs, including LPS, phosphatidylethanolamine, phosphatidylglycerol, and cardiolipin, which are closely related to membrane curvature [89, 90]. Importantly, some specific lipid species were also found to be selectively enriched in BEVs, and lipid analysis data showed that the content of phosphatidylglycerol and stearic acid, which are involved in membrane fluidity and rigidity, present in BEVs was significantly higher than that in the cellular outer membrane [56, 91]. Another study on *Listeria monocytogenes* showed that unsaturated fatty acids, including phosphatidylethanolamine and sphingolipids, were more abundant in BEVs, while glycoglycerolipids were relatively underexpressed, indicating that the lipid composition of BEVs is not uniform across bacterial species [92]. Furthermore, it has been revealed that *P. gingivalis* BEVs also possess unique properties in terms of LPS composition [60]. Therefore, the lipid composition of BEVs may vary depending on the bacterial species and environmental conditions that are adapted to their biological functions.

***Nucleic acids***. It has been well established that BEVs can incorporate bacterial genetic material, including DNA and, more recently, different types of RNAs [93]. BEVs allow the delivery of DNA into other bacterial cells. Both chromosomal and plasmid DNA have been detected in the lumen or surface of BEVs derived from bacteria such as *E. coli, Neisseria gonorrhea*, *P. gingivalis*, *Acinetobacter baumannii*, and *Pseudomonas aeruginosa* [70, 94–96]. Although there is a limited understanding of its biological functions, evidence suggests that BEV‐bound DNA may play a role in biofilm formation, immunoregulation, adhesion, and virulence [96–98]. Previous studies have well demonstrated that BEVs contain abundant RNAs, including messenger RNA (mRNA), transfer RNA (tRNA), ribosomal RNA (rRNA), and small RNA (sRNA), of which a large proportion of these BEV sRNAs and noncoding RNAs are from uncharacterized intergenic regions and may be responsible for regulating gene expression in target cells [47, 96, 99]. Recent evidence has shown that mRNAs in BEVs may be translated to produce microbial proteins in target cells, and sRNAs in EVs may interact with specific target cell factors to attenuate or enhance gene expression via transcription, mRNA processing, and translation [100]. Vast numbers of BEV‐associated RNAs have been validated by RNA profiling analysis, but their functions remain largely unclear [47]. Several studies have reported that the delivery of BEV RNAs to host cells may participate in the regulation of inflammation, immune response, infection, and chemotherapeutic resistance [101, 102]. Overall, abundant coding and regulatory nucleic acids have been well verified to be enriched in BEVs, yet their exact composition and function require further investigation.

As mentioned above, the BEV's cargo composition is highly heterogeneous and largely depends on its origin, release location, and physiological or pathological state. Although considerable research has been conducted to elucidate the potential mechanisms that confer selective enrichment of proteins, lipids, and nucleic acids in BEVs, the exact sorting mechanisms are still unclear [103–105]. One proposed mechanism for selective cargo delivery into BEVs involves outer membrane lipid chaperones. Both neutral (A‐band LPS) and negatively charged (B‐band LPS) O antigen residues are present on the outer membrane; only B‐band LPS is found to be associated with BEVs. In BEVs released by the B‐band LPS mutant strain of *P. gingivalis*, the enrichment process of virulence factors such as gingipains was apparently abolished, suggesting that outer membrane lipid chaperones with high A‐LPS composition may play a critical role in selective cargo packaging due to their affinity for the overall charge [59, 60]. Consistently, a comprehensive analysis of BEV cargoes of group A *Streptococcus* identified that the lipid composition of the bacterial membrane was responsible for the selective enrichment of specific proteins and RNA species [47]. Besides, a recent study on *Staphylococcus aureus* characterized the BEVs' protein composition and revealed that the main protein cargoes were positively charged, constituting more small residues and fewer aromatic and aliphatic groups compared with the whole‐cell proteome, which further implicated the role of physicochemical properties (and potential charge) in cargo sortation [104]. Furthermore, in contrast to the listed passive sorting mechanisms, researchers have speculated alternative active sorting mechanism that the membrane convex or concave topological domain shaped during BEV budding may selectively enrich specific complementary molecules and guide them into BEVs [106].

## BEVs BIOLOGICAL FUNCTIONS

Since the discovery of BEVs, research in the field has predominantly focused on their contributions to a wide spectrum of physiological and pathophysiological conditions. After detachment from the parent cells, BEVs translocate and interact with the cell surface of recipient cells subsequently transferring cargoes to the cytoplasm, thereby triggering downstream signaling pathways. To date, it has been well demonstrated that BEVs play an important role in a broad spectrum of biological events, including biofilm formation, antibiotic resistance, delivery of virulence factors, immunomodulation, and inflammation [107–110]. In this section, we mainly focus on recent advances in the field of bacteria–host interactions.

### Defensive stress response

BEV secretion serves as a conservative stress response strategy and has been implicated in diverse unfavorable environmental conditions, such as heat shock, nutrient deficiency, pH changes, temperature extremes, and the presence of antibiotics [111]. Generally, multiple direct and indirect mechanisms regarding the protective role of BEV secretion against stressors have been introduced, including promoting biofilm formation, exporting misfolded aggregates, and decoying membrane‐targeted attacks [112].

Biofilms are essential and complex structures that enable bacteria to thrive in a protected microenvironment and are deeply involved in bacterial growth, resistance, and survival [113]. The effects of BEVs on biofilm formation could be positive or negative, based on differences in bacterial species and physical state. Notably, BEV cargoes are suggested to be fundamental constituents of biofilms; for instance, environmental DNA forms a key biofilm substrate, and adhesion‐related proteins confer adhesive properties. Moreover, many biofilm matrix proteins, such as leucine aminopeptidase PaAP, have been identified in BEVs derived from *P. aeruginosa*, *V. cholerae*, and *Helicobacter pylori* [114–116]. BEVs released in biofilms can also sequester antibiotics, thereby promoting bacterial survival and biofilm development. Evidence from *Aeromonas* strains and *Enterobacter cloacae* indicates that even the quantity and concentration of BEVs are positively correlated with the formation of biofilms [117, 118].

Given the fact that BEVs are spherical nanostructures containing bacterial membrane components, outer membrane proteins, lipids, and soluble materials bound to the external surface, which confer the potential for BEVs to act as decoys, enabling bacteria to evade external attacks [119]. For instance, phage treatment serves as an efficient alternative intervention to limit bacterial infection, which depends largely on the presence of phage receptors on the outer membrane surface. During phage treatment of *V. cholerae*, secreted BEVs can act as natural decoys to defend bacteria from phage predation and weaken the outcome of phage treatment [120]. T4 is a well‐studied bacteriophage, and there is an obvious binding and reduction of infection when T4 is co‐incubated with BEVs, allowing enterotoxigenic *E. coli* (ETEC) to escape many lethal environmental stresses [121]. Thus, BEV production may effectively enhance survivability by neutralizing environmental attacks that target parent bacteria.

Moreover, BEVs have been suggested to play an essential role in antimicrobial resistance by harboring neutralized enzymes or directly sequestering antimicrobials away from bacteria [122]. BEV production is positively linked to antibiotic treatment, and analysis of the proteome composition of BEVs revealed the presence of multiple antibiotic‐degrading enzymes, including β‐lactamase, indicating a strong relationship between BEV production and antibiotic resistance [123, 124]. BEV‐mediated absorption can help bacteria fight antibiotics. An obvious decline in the therapeutic efficiency of antimicrobial peptide (AMPs) polymyxin B and colistin in *E. coli* was observed with the supplementation of BEVs or in a hyper‐vesiculating mutant of *E. coli* [121]. Blocking BEV release can increase bacterial susceptibility to antibiotics [125]. In addition, adaptive mechanisms involving intra‐ and inter‐species transfer of functional antibiotic enzymes and transporters to remove antibiotics have also been reported to reduce the efficacy of antibiotics and contribute to bacterial virulence [126].

### Delivery of virulence factors

As mentioned above, intensive research has recognized the critical role of BEVs as vehicles to deliver virulence effectors to host cells, allowing pathogenic bacteria to access distal sites within the body without directly translocating from their environmental niche [127]. Generally, virulence factors originate from the BEV lumen and membrane structure, including toxins and adhesins. Cargo inclusion into BEVs offers several distinct advantages over other delivery strategies, such as immune system evasion, protection from proteolytic degradation, increased concentration and stability, long‐distance delivery, and host‐cell targeting specificity [127, 128]. Proteomic analysis of BEV compositions has revealed the presence of diverse pathogenic virulence factors, including leukotoxin, Shiga toxin, hemolysin, genotoxins, *β*‐lactamase, and active AmpC [127, 129, 130]. For example, *H. pylori* is the most important contributor to the development of chronic inflammation and peptic ulceration, *and H. pylori*‐derived BEVs containing oncogenic virulence factor cytotoxin‐associated gene A (cagA) result in increased epithelial permeability and disrupt the integrity of epithelial barriers [131]. Similarly, gingipain is the typical toxin released by *P. gingivalis* that causes severe damage to the supporting tissues of teeth and periodontitis, while its virulence was observed to be enhanced by the synergistic effect of BEVs [132]. The cytotoxicity of purified *Mycobacterium ulcerans* BEVs containing the toxin mycolactone is obviously amplified compared to that of purified toxin alone, indicating the pivotal role of toxin‐associated BEVs in bacterial pathogenicity [133]. Furthermore, BEV‐mediated long‐distance delivery of bacterial proteins to host cells may represent a general pathogenic pattern observed for multiple pathogens [134, 135].

### Pro‐ and anti‐inflammatory responses

Inflammation is an adaptive response by the host to ensure removal of detrimental stimuli and promote damage tissue repair, which is usually timely terminated upon restoration of tissue homeostasis, and excessive inflammation (termed a cytokine storm) can be harmful to the body. All structural and functional components of BEVs originate from bacteria, such as LPS, lipoproteins, and toxins, and it is not surprising that BEVs contribute to the onset and progression of inflammation in the host. Although BEV‐associated inflammation is known to be present in diverse host tissues, the mechanisms involved have not been extensively researched [136]. Mounting evidence suggests that pattern recognition receptors (PRRs) expressed in host immune cells are responsible for sensing and recognizing BEVs via pathogen‐associated molecular patterns (PAMPs). Different PRR family members have been identified based on their localization, including Toll‐like receptors (TLRs), C‐type lectin receptors (CLRs), retinoic acid‐inducible gene (RIG)‐I‐like receptors (RLRs), and NOD‐like receptors (NLRs) [136]. PRR activation signals transduced by common adaptor proteins lead to upregulation of genes, transcription factors, and kinases involved in inflammatory responses. As the loading content and composition of BEVs vary among bacterial species, the extent of the proinflammatory response can be different. For example, THP‐1 cells exposed to *Filifactor alocis* showed increased expression of various chemokines, such as C‐C Motif chemokine ligand 1 (CCL1), CCL2, CCL5, C‐X‐C motif chemokine ligand 1 (CXCL1), CXCL10, intercellular adhesion molecule 1 (ICAM‐1), interleukin‐6 (IL‐6), and IL‐8 [137], whereas *Streptococcus pneumoniae*‐secreted BEVs elicited enhanced production of IL‐6, IL‐8, IL‐10, and tumor necrosis factor (TNF) in human monocyte‐derived dendritic cells [138]. NOD‐like receptor thermal protein domain associated protein 3 (NLRP3) inflammasome complex activation is responsible for the maturation of caspase‐1 and subsequent secretion of mature IL‐1 family cytokines, including IL‐1β and IL‐18. Recent studies have reported that PG containing BEVs released by mucosal pathogens *H. pylori* and *P. aeruginosa* could be specifically detected by the NOD1 receptor, resulting in NF‐κB pathway‐dependent activation of NLRP3 inflammasome and IL‐1β release [139–141]. Collectively, these studies highlight that there are a broad range of mechanisms by which BEVs mediate the induction of proinflammatory responses in the host.

Apart from the proinflammatory effects of pathogenic bacteria‐derived BEVs mentioned above, the anti‐inflammatory activity of BEVs has also been described in various nonpathogenic species, mostly commensal and probiotic bacteria. The decreased abundance of the *Lactobacillus* population is a typical probiotic that is tightly involved in the onset and progression of inflammatory gastrointestinal diseases; in LPS‐ and dextran sulfate sodium (DSS)‐mediated inflammation in vitro and in vivo, *Lactobacillus*‐derived BEVs showed a marked anti‐inflammatory effect by inhibiting cyclooxygenase‐2 (COX‐2), inducible nitric oxide synthase (iNOS) and nuclear factor‐κB (NF‐κB) expression [142]. Similarly, *Lactobacillus*‐derived BEVs can also reduce interferon gamma (IFN‐γ) production and dampen proinflammatory cytokine release in a monocyte‐dependent manner [143]. Another study utilized *E. coli* Nissle 1917 (EcN) purified BEVs to treat DSS‐induced colitis in a mouse model, and the results showed that pretreatment with BEVs significantly attenuated DSS‐induced weight loss, clinical symptoms, and histological scores [144]. Furthermore, nucleic acids packaged inside BEVs also regulate inflammatory responses. sRNA52320, a small sRNA detected in *P. aeruginosa*‐secreted BEVs, was found to downregulate LPS‐induced cytokine release via the mitogen‐activated protein kinase (MAPK) signaling pathway [145]. Collectively, the contribution of BEV‐mediated inflammation to host benefits and pathogenicity has been well illustrated in the literature, while emerging questions regarding the nature of these components and the underlying mechanisms in host–pathogen interactions have yet to be fully answered.

### Immunomodulation

To date, there have been extensive studies aimed at exploring the role and underlying mechanisms of BEVs in the occurrence and development of various human pathologies, of which BEV‐associated immunological balance has attracted much research interest. Generally, BEVs can confer immunosuppressive or immunostimulatory roles depending on the active components within the EVs and the distinct characteristics of diverse diseases. Mounting evidence has established that BEVs tightly interact with a range of innate and adaptive immune cells [18].

Monocyte‐macrophages are the most widely investigated innate immune cells responsible for host tissue surveillance as well as rapid detection and elimination of invasive pathogens. In cases of infection caused by *P. gingivalis, Treponema denticola*, *Legionella pneumophila*, and *Salmonella typhimurium*, secreted BEVs can interact with and activate monocytes‐macrophages, resulting in the release of cytokines, including nitric oxide (NO), TNF‐α, IFN‐α, IL‐6, IL‐8, and IL‐1β [140, 146]. *H. pylori*‐derived BEVs have also been shown to reduce cytokine responses by promoting IL‐10 production in human peripheral blood mononuclear cells [147]. In terms of the regulatory influence of BEVs on neutrophils, it is suggested that purified BEVs can directly interact with and activate neutrophils, as demonstrated in *S. aureus* [148]. Neutrophil extracellular traps (NET) are specific neutrophil‐related antimicrobial mechanisms, and *S. pneumoniae* BEVs packaged with extracellular DNase TatD may subvert immune responses by disrupting NET formation, thereby modulating the host's response to infection [149]. Dendritic cells (DCs) are antigen‐presenting cells (APCs) that bridge innate and adaptive immune systems. DCs stimulated by *S. typhimurium*‐derived BEVs displayed increased surface expression of MHC‐II and CD86 and inflammatory mediators (NO, TNF‐α, and IL‐12), compared to levels induced by bacterial cells, indicating the potent proinflammatory and antigenic properties of BEVs [150]. Interestingly, in the case of the human commensal *Bacteroides fragilis* regulating the human immune response, purified BEVs delivered to intestinal dendritic cells exhibit anti‐inflammatory effects by promoting IL‐10 production and reducing IL‐17A expression from CD4+ Foxp3+ regulatory T cells [151]. In summary, these studies demonstrated that the outcome of BEV and DC interactions is largely dependent on the microbial species and host physiological state.

In addition to the aforementioned immunomodulatory effects of BEVs on innate immune cells, BEVs have also been shown to modulate the adaptive immune response of the host. *Moraxella catarrhalis*, a respiratory pathogen residing in the tonsils, is readily endocytosed and eliminated by human tonsillar B cells. However, BEVs containing DNA have the potential to interact with and activate the B cell TLR9 signaling pathway; thus, BEVs serve as a decoy to ultimately favor bacterial survival [152]. In addition, combinations of BEVs from *Neisseria meningitidis* serogroup A and W_135_ showed the ability to induce an IgG2a/IgG2b isotype profile and IFN‐γ production, suggesting the production of long‐term B memory cells and activation of cell‐mediated immunity [153]. Nevertheless, more evidence regarding how BEVs directly interact with B cells and the underlying mechanisms is still needed. By contrast, T cell responses elicited by BEVs have been illustrated in a broad spectrum of pathogenic species, such as *S. aureus*, *Francisella tularensis*, and *S. typhimurium* [154, 155]. In adoptive transfer and gene‐knockout research, *E. coli*‐derived BEV immunization effectively stimulated T cell immunity and systemic inflammatory response syndrome primarily via Th1 and Th17 cell responses, ultimately preventing bacteria‐induced lethality [156]. BEVs can also exhibit an immunosuppressive role in T‐cell responses. The addition of *H. pylori*‐derived BEVs to peripheral blood mononuclear cells increased the expression of COX‐2, PGE2, and IL‐10, accompanied by the subsequent inhibition of T cell proliferation [157]. Thus, BEVs may possess versatile immunomodulatory roles in T cell responses in the host, based on their origin.

## BEVs IN PATHOGENESIS

Over the past decades, with the rapid advancement of detection and analytical techniques, mounting evidence has demonstrated the pivotal role of BEVs in the initiation and progression of a wide spectrum of human pathologies, including gastrointestinal, respiratory, dermatological, neurological, periodontal, metabolic, and musculoskeletal diseases, and cancer.

***Gastrointestinal disease***. Compared to other organs, the gastrointestinal tract harbors the largest number and variety of microbes that play essential roles in energy acquisition, metabolism, and immune regulation. BEVs released into the gut lumen have the potential to cross the mucus layer and mediate cross–kingdom interactions with host cells, which are strongly implicated in host health. Several studies have confirmed the pathogenic role of BEVs in various gastrointestinal disorders such as peptic ulcers, enteric infections, and inflammatory bowel disease (IBD) [158]. *H. pylori* colonization of the human stomach has been well established as the principal etiologic agent of peptic ulcer disease. On the one hand, *H. pylori* BEVs containing several virulence factors (VacA, urease, and CagA) are readily internalized, leading to impaired bioactive function and disruption of tight junctions in gastric epithelial cells [86, 159]. On the other hand, *H. pylori* BEVs may contribute to proinflammatory effects on epithelial and immune cells by promoting IL‐6, IL‐8, TNF‐α, and IFN‐α production [160]. *V. cholerae* and pathogenic *E. coli* are representative etiologic agents implicated in gastroenteritis. BEVs secreted from *V. cholerae* and pathogenic *E. coli* serve as carriers for many virulence factors such as cholera toxin, heat‐labile enterotoxin, hemolysin, and Shiga toxin, resulting in endothelial cytotoxicity, apoptosis, and inflammatory cytokine release [161, 162]. IBD, which comprises Crohn's disease and ulcerative colitis, is characterized by chronic relapsing‐remitting inflammation of the gastrointestinal tract. Alterations in intestinal microbial homeostasis are closely associated with the pathogenesis and development of IBD [163]. BEVs derived from pathogenic *E. coli* are found to stimulate inflammatory production via the TLRs pathway in intestinal epithelial cells, and even induce endothelial and epithelial mitochondria‐associated apoptosis via toxin delivery [164–166]. Despite the above facts, there are still many unanswered questions concerning the regulatory mechanisms of BEV‐mediated microbe–host interactions.

***Respiratory disease***. Numerous studies have characterized the respiratory microbiome in human health and disease. An imbalance in the respiratory microbiome is associated with a broad range of lung diseases, including chronic obstructive pulmonary disease (COPD), pulmonary fibrosis, asthma, and acute lung injuries [167]. Chronic airway inflammation is a common feature in multiple chronic lung diseases, including asthma and COPD. *S. aureus* colonization of the nasopharynx may be a risk factor for lung disease. Kim et al. have provided new evidence that the addition of *S. aureus* BEVs to macrophages and airway epithelial cells results in the production of IL‐6 and TNF‐α, and repeated airway exposure with *S. aureus* BEVs can induce Th1 and Th17 neutrophilic pulmonary inflammation in a TLR2‐dependent manner [168]. Similar outcomes were observed in BEVs that were mainly derived from indoor dust Gram‐negative bacteria such as *E. coli* [80, 169]. Furthermore, under conditions of microbe‐associated acute lung injury, BEVs containing PAMPs and damage‐associated molecular patterns (DAMPs) are known to trigger inflammatory cell (macrophage and neutrophil) infiltration and cytokine (IL‐6, TNF‐α, and IL‐1β) expression in lung tissues [170].

***Dermatological disease***. Acne vulgaris (acne) is a highly prevalent inflammatory skin condition that involves the microbiome and its interactions with the innate immune system. A microbial imbalance of skin bacteria including *Cutibacterium acnes*, *Staphylococcus epidermidis*, and *S. aureus* has been implicated in the pathophysiology of inflammatory acne [171]. Keratinocytes are the main cell type of the epidermis and participate in the activation of inflammation and immune responses when exposed to DAMPs and PAMPs. Proteomic analysis identified multiple virulence factors (such as NlpC/P60, CAMP factor, and Hta domain protein) in *C. acnes*‐derived BEVs [172]. Meanwhile, *C. acnes*‐derived BEVs significantly induced IL‐8 and GM‐CSF production and dysregulated epidermal differentiation in epidermal keratinocytes, leading to acne‐like phenotypes [173]. Atopic dermatitis, characterized by epidermal thickening and infiltration by eosinophils and mast cells, is intrinsically associated with abnormal microbiome homeostasis in the skin, especially *S. aureus*. A previous metagenomic analysis together with serum detection of pathogen‐specific EVs in atopic dermatitis patients showed that *S. aureus* is the most dominant microbe detected in atopic dermatitis lesions, and its EV‐specific IgG and IgE were twofold higher in atopic dermatitis patients than in normal controls [174]. Histological analyses observed abundant staphylococcal protein A (SPA) in the epidermis of atopic dermatitis lesions, and proteomic analysis revealed that *S. aureus* BEVs contain various pathogenic molecules (such as a‐hemolysin and cysteine protease), indicating its potential links with epidermal barrier dysfunction [175, 176]. Moreover, intact *S. aureus* BEVs stimulated the release of proinflammatory cytokines (including IL‐1b, IL‐6, IL‐8, and MIP‐1a) in keratinocytes in a TLR2‐ and NOD2‐dependent manner. Topical application of intact *S. aureus* BEVs specifically induced infiltration of inflammatory cells and inflammatory responses in atopic dermatitis‐like skin lesions in an in vivo model [176]. Thus, *S. aureus* BEVs may be potential therapeutic targets for the management of atopic dermatitis aggravation.

***Neurological disease***. Emerging evidence has demonstrated the critical role of microbiota in bidirectional communication between the gut and the brain, and it is not surprising that BEVs may participate in the pathogenesis and progression of many neurological conditions [177]. The blood–brain barrier (BBB) is a highly selective barrier that controls molecular and cellular trafficking between the bloodstream and neural tissue. Several studies have provided evidence for the presence of bacterial components, such as rRNA and rDNA, in brain tissues, and it has been speculated that a fraction of these microbial nucleic acids may cross the BBB through BEVs delivery [178, 179]. Moreover, BEVs can disrupt BBB integrity and result in increased access of other BEVs or bacterial products to the brain [180, 181]. Neuroinflammation is a common pathological feature that contributes to abnormal protein aggregation, neuronal dysfunction, and even cell death in multiple neuropsychiatric disorders including Alzheimer's disease (AD), Huntington's disease (HD), Parkinson's disease (PD), and depression/anxiety. On the one hand, once translocated into the brain, BEVs directly alter neurologic function and induce pathology through releasing their biological cargoes, such as enzymes and regulatory sRNAs [181, 182]. *Aggregatibacter actinomycetemcomitans* BEVs ferry sRNA across the BBB to increase brain TNF‐α levels and subsequent neurologic effects via TLR8 and NF‐κB signaling pathways [182]. By contrast, BEVs containing various DAMPs/PAMPs (such as LPS, lipoprotein, and nucleic acids) readily stimulate host immune and nonimmune cells via TLR and NOD signaling pathways to produce systemic inflammation, ultimately resulting in neuroinflammation [183]. Thus, BEVs may represent an important alternative pathway in the pathogenesis of neurological disorders.

***Periodontal disease***. Periodontal disease is a common infectious disease that involves a range of bacterial species and is characterized by a surrounding inflammatory microenvironment and destruction of periodontal structures. BEVs derived from *P. gingivalis* have been implicated in the formation of oral inflammatory microenvironments. As early as 1985, researchers observed the phenomenon of MEVs produced by *P. gingivalis*, but their pathogenic role was not defined [184]. Proteomics and RNA‐seq analyses have revealed that *P. gingivalis* BEVs are enriched in diverse virulence factors or modulators of virulence factors, including gingipains, heme‐binding lipoproteins, and noncoding RNAs, enabling bacteria to penetrate deep tissues and activate an inflammatory host response [185]. Compared with the parent bacteria, BEVs showed amplified production of cytokines and proapoptotic factors (including TNF‐α, IL‐6, IL‐8, IFN‐γ, LDH, and 7‐AAD) in macrophages and endothelial cells, leading to cellular impairment, connective tissue destruction, and alveolar bone resorption [128, 186, 187]. In addition, recent understanding of the constituents of BEVs released by *A. actinomycetemcomitans*, *Filifactor alocis*, and *Fusobacterium nucleatum* also provides clues for deciphering the pathogenic role of BEVs in periodontal disease [84, 137, 188, 189].

***Metabolic disease***. Mounting evidence has established the central role of the microbiome in host energy homeostasis and its alterations have strongly been linked to the onset and progression of multiple metabolic disorders, especially obesity and diabetes [190–192], although the exact mechanisms remain unclear. In this regard, BEVs are postulated to be key mediators of microbiota–host communication by encapsulating a wide range of bioactive molecules. *Akkermansia muciniphila* is a beneficial bacterium that modulates intestinal barrier integrity. *A. muciniphila* EV administration to a high‐fat diet (HFD)‐induced diabetic mice resulted in decreased gut barrier permeability, reduced body weight gain, and improved glucose tolerance. Moreover, there are fewer *A. muciniphila* EVs in the fecal samples of patients with type 2 diabetes (T2D), indicating a positive relationship with energy metabolism [193]. In addition, obesity and insulin resistance are often associated with low‐grade inflammation, with adipocytes and macrophages being master players. TLR‐4 dependent cytokine production is an important pathway for BEV‐mediated proinflammatory tissue; TLR‐4 overexpression has been detected in adipose tissue, skeletal muscle, and liver [194, 195]. Under HFD conditions, TLR4 depletion‐mice showed decreased blood glucose and glucose tolerance and improved insulin sensitivity. Furthermore, *Pseudomonas panacis* BEVs were reported to block the insulin‐signaling pathway in both skeletal muscle and adipose tissue and induce a typical diabetic phenotype in mice [196]. Moreover, BEVs derived from *P. gingivalis* were also introduced to participate in the progression of T2DM, and BEVs containing the active protease gingipains were translocated to the liver in mice, which resulted in changes in glucose metabolism and reduced insulin sensitivity. *P. gingivalis* BEVs also attenuated insulin‐induced Akt/GSK‐3β signaling in hepatic HepG2 cells [37, 197]. These results revealed a complex and multifaceted relationship between BEVs and host metabolism.

***Musculoskeletal disease***. Bone homeostasis is dynamically maintained by the balance of bone formation and bone absorption, and alterations in intestinal microbiota composition and metabolites are implicated in impaired bone metabolism by affecting mineral absorption and stimulating inflammation [198, 199]. Abnormal bone loss can be caused by aging, hormonal imbalance, and dietary factors, all of which have been documented to be accompanied by microbiome changes [41, 200–203]. In addition, a recent study by Liu et al. reported that gut microbiota from healthy children alleviated bone loss and altered bone metabolism in ovariectomy‐mice by reversing the ovariectomy‐induced reduction of *A. muciniphila*. Notably, the protective effects of *A. muciniphila* treatment against osteoporosis are largely attributed to the secretion of BEVs, which translocate and accumulate in bone tissues to promote osteogenic activity and inhibit osteoclast formation [204]. Similarly, Chen et al. also revealed that the composition of *Lactobacillus animalis*, a well‐studied probiotic strain, was reduced in mice with glucocorticoid‐induced osteonecrosis of the femoral head (ONFH), and oral supplementation with *L. animalis* significantly mitigated the extent of ONFH by promoting vascularization, osteogenesis, and suppressing cell apoptosis. Mechanistically, *L. animalis*‐BEVs can selectively ferry various functional proteins and deliver them to the femoral head, whereby BEVs prevent trabecular bone damage and bone loss by improving the activity and function of endothelial and bone cells [205]. Additionally, it has been demonstrated that a shift in microbiota is identified as a key risk factor influencing the pathogenesis of rheumatoid arthritis (RA), which is a chronic inflammatory disease characterized by synovial inflammation, leading to progressive cartilage and bone destruction [201]. *P. gingivalis* is an important pathogenic bacterium linked to RA, and DNA derived from *P. gingivalis* has been detected in the synovial fluid of patients with RA [206]. Peptidylarginine deiminase (PAD), an enzyme in *P. gingivalis*, is reported to mediate the citrullination of human proteins and potentially contribute to the loss of tolerance to citrullinated proteins in RA. Gabarrini et al. found that PPAD can be modified by A‐LPS and is associated with BEVs escaping proteolytic degradation [207]. Thus, BEV‐dependent regulatory mechanisms may represent alternative pathways for maintaining bone homeostasis.

***Cancer***. Alterations in the diversity and function of microbiome homeostasis are associated with the pathogenesis of diverse tumors. BEVs detached from the outer membrane can enter the circulatory system to disseminate to distant organs and tissues, possibly contributing to tumorigenesis. There are some reasonable tumor‐promoting mechanisms of BEVs, including direct impact on genomic stability or cell cycle, generation of a proinflammatory tumor microenvironment, and production of certain carcinogens [208]. As mentioned above, TLRs are typical PPR‐sensing BEV components, including nucleic acids, LPS, and peptidoglycans, and trigger temporal inflammation to ensure pathogenetic clearance from the body, while uncontrolled chronic inflammation also results in disease, including cancer [209]. For instance, metagenomic analyses have shown enrichment of *F. nucleatum* in colorectal carcinoma tissue, and BEVs released by *F. nucleatum* may drive oncogenesis by suppressing the immune response and eliciting inflammation [210, 211]. Similarly, *H. pylori*‐derived BEVs were found to be upregulated in the gastric juice of patients with gastric cancer. *H. pylori*‐derived BEVs containing CagA and VacA are suggested to hold capabilities of alteration of cell growth, damage to DNA, and aneuploidy, implying its tight association with gastric cancer [102]. Additionally, *P. gingivalis*, the major pathogen in periodontal disease, was recently shown to promote the progression of oral squamous cell carcinoma (OSCC). *P. gingivalis* BEVs are packaged with diverse sRNAs with the potential to target host mRNA function and/or stability, of which sRNA23392 was found to promote invasion and migration of OSCC cells [212]. To date, various lines of evidence have shown that the impact of BEVs on oncogenesis is largely context‐dependent and the potential role of BEVs in tumorigenesis are well investigated in multiple studies [102, 213–215]; however, the underlying mechanisms are still not fully elucidated.

## BEVs IN CLINICAL APPLICATION

**Figure 4:**
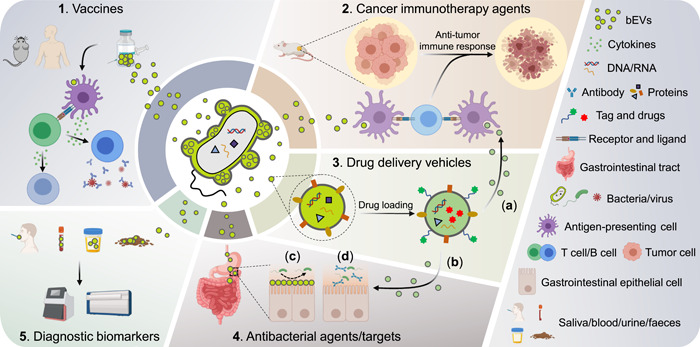
BEVs clinical applications at a glance. BEVs have emerged as candidates with strong clinical potential, including utilization in vaccine development, disease diagnoses, and novel therapeutics. Reprinted with permission [21]. Copyright 2020, Elsevier. BEV, bacterial extracellular vesicle.

Based on the distinct features of BEVs for cost‐effectiveness in production, stability to transport and storage, convenience for modification, and proven immunomodulatory properties, BEVs have emerged as candidates with strong clinical potential, including application in disease diagnoses, vaccine development, and novel therapeutics (Figure 4).

### BEVs in diagnostic analytics

As discussed above, an imbalance in microbiome homeostasis has been strongly associated with the onset and progression of a wide range of human pathologies, and disease‐associated microbiome changes may be reflected in the abundance and composition of BEVs in biofluids [216]. BEVs released by microbiota can directly penetrate the mucosa and adjacent epithelium, reach the bloodstream, and subsequently travel throughout the body [217]. BEVs packaged with diverse parental genomic and metabolic information indicate their origin and reflect the composition and metabolic status of the microbiota. In this context, it is desirable to detect BEVs profiles in the urine, blood, and other bodily fluids to assist in the diagnosis of physiological and disease states.

In recent years, advanced and accurate meta‐omics technologies (metagenomics and metabolomics) combined with bioinformatics analytic technologies have provided a detailed landscape of the phylogeny, interactions, and functions of BEVs in health and disease conditions. In contrast to human cell‐derived EVs, the exploration of BEVs as biomarkers in disease is still in its infancy. To date, several BEV‐based disease diagnosis models have been developed for Alzheimer's [218], atopic dermatitis [219], allergies [220], respiratory diseases [221], and cancer [222]. Despite these encouraging results, a variety of challenges still need to be addressed to facilitate clinical adoption. One of the issues is the requirement for a special technological platform to collect, screen, and interpret large datasets. In addition, the actual presence of BEV extracts is highly complex and depends on their source and isolation approaches, which largely weakens the sensitivity and specificity of BEVs' diagnostic efficiency.

Notably, BEV‐based diagnostic models integrated with other diagnostic modalities can provide improved diagnostic performance. A combination of metagenomic and metabolomic analyses of BEVs isolated from the feces of patients with colorectal cancer and healthy participants demonstrated an association between microbial changes and metabolic alterations, indicating that BEVs carry a dynamic range of metabolic information reflecting the host's nutritional state, metabolism, and immune responses in the presence of disease [223]. Furthermore, BEV‐metagenomics and immunoassays can be used to develop a novel diagnostic methodology. For example, exposure to BEVs in indoor dust is involved in the pathogenesis of respiratory diseases, and the combination of BEVs metagenomic characteristics assessment and ELISA‐based analysis of serum IgGs against core pathogenic indoor dust BEVs was found to be an effective diagnostic tool for lung disease [224]. Therefore, BEV‐based diagnostic technologies have the potential to be widely applied in the clinical setting.

### BEVs in vaccine design

Rapid spread of antibiotic resistance has provoked an urgent need for novel antimicrobial therapeutics, including vaccine formulations. BEVs are nonreplicating entities, with a size of 20–250 nm, and incorporate multiple PAMPs mimicking the immunogenic properties of the producing bacteria, which has increased the appeal of BEVs as attractive vaccine candidates [225]. In recent years, the potential of BEVs as vaccines against bacterial pathogens has been extensively investigated [226, 227].

As discussed before, BEVs are primarily composed of bacterial outer membrane constituents and thus contain key antigenic components, such as LPS, lipoproteins, and nucleic acids, to stimulate humoral and cellular immune responses. The intrinsic features of BEVs have been exploited in the field of vaccinology for decades. The most representative and successful BEV vaccine against *Neisseria meningitidis* was first licensed for use in Cuba as early as 1987 and subsequently employed as a vaccine to combat the outbreak of *meningococcal* group B disease in Norway, New Zealand [227]. Similarly, BEVs derived from many other Gram‐negative and Gram‐positive bacteria are being developed as candidate vaccines against pathogenic infections caused by, *V. cholerae*, *Klebsiella pneumoniae*, *Bordetella pertussis*, *S. typhimurium*, *S. aureus*, *S. pneumoniae*, and *Clostridium perfringens*. BEVs vaccines have immunostimulatory efficiency comparable to that of the whole inactivated cell vaccine, and animals vaccinated with BEVs displayed cell‐mediated and humoral immune responses, resulting in decreased bacterial burden and lethal challenge [228–231]. These studies highlight the potential of BEVs in vaccine development.

In addition, BEVs can be developed as adjuvants to enhance and shape the quality and quantity of immune response. BEVs contain multiple PAMPs (such as LPS and lipoproteins) in their native conformations that interact with the corresponding PPRs (such as TLRs and NLRs), promoting APCs maturation and antigen‐specific immune response activation [216]. For instance, recent studies have reported that coimmunization *of E. coli* BEVs with malaria transmission‐blocking antigens in mice elicited robust humoral and cellular immune responses, and antibody titers were found to be comparable to conventional adjuvants, such as cholera toxin and MF59C.1, accompanied by no significant side effects or weight loss [232]. Similarly, BEVs also display strong adjuvant activity against influenza vaccine antigens and hepatitis B virus surface vaccine antigens [233, 234]. Intranasal injection of the influenza vaccine with BEVs acquired from a combination of ammonium sulfate precipitation and DGC, significantly stimulated antigen‐specific humoral and cellular immune responses in mice, leading to a significantly higher protection rate against challenge with a lethal dose of homologous or heterologous influenza viruses without adverse effects [233]. These results demonstrate the potential of BEVs as safe and effective adjuvants for vaccine utilization.

Despite these promising data, several challenges must be addressed before BEVs can safely be used for vaccination, such as their high reactogenicity, heterogeneity, strain variation, and low expression of relevant protective antigens [227]. Genetic engineering techniques are under investigation to address these challenges by improving BEVs immunogenicity, safety, and production while removing BEVs toxins and deleterious compounds. Genetic manipulation of the parent bacteria can refine the functionality of BEVs; for instance, heterologous antigens can be anchored to native proteins located on the surface of BEVs, including the cytolysin A protein of *E. coli* or factor H binding protein (fHbp) of *N. meningitidis*, resulting in their association with OMVs at high concentration [235–237]. These engineered BEVs based vaccines have been successfully validated against pathogens in models of infection with H1N1 influenza, *A. baumannii*, and *S. pneumoniae* [236–238]. Furthermore, the issue of low yield efficiency that restricts the use of BEVs as potential vaccines is largely resolved by genetically engineered approaches to alter the structure and integrity of the bacterial outer membrane in hypervesiculating bacteria [235, 238]. However, it remains unclear whether these modifications to the bacterial outer membrane result in distinct characteristics of BEVs compared with their wild‐type counterparts.

### BEVs in therapeutics development

In recent years, precision medicine, also known as personalized medicine, has attracted increasing research interest, which enables the individualization of patient care from diagnosis to therapy. Given the inherent structural and functional characteristics of BEVs, such as nanoscale size, cell‐free system, immunogenicity, and ease of genetic engineering editing, BEVs have shown great therapeutic potential in precision medicine, and their clinical applications have been widely explored.

Therefore, native or natural BEVs derived from commensal bacteria and probiotics may serve as potential therapeutic candidates. For example, *L. animalis* reduction has been associated with glucocorticoid‐induced osteonecrosis of the femoral head. Administration of *L. animalis* BEVs greatly improved bone microarchitecture and mitigated the extent of osteonecrosis by enhancing blood vessel abundance, increasing osteogenic activity, and reducing apoptosis [205]. Direct replenishment of *A. muciniphila* BEVs has also been shown to restore multiple pathological events, including rebalancing bone metabolism to prevent osteoporosis, improving glucose tolerance and obesity, and enhancing epithelial function to relieve IBD [193, 204, 239]. In addition, treatment with *Lactobacillus plantarum* BEVs showed therapeutic effects on stress‐induced depression‐like behaviors by altering the expression of neurotropic factors in the hippocampus of mice [240]. Therefore, BEV‐based therapeutic strategies have emerged as novel and promising treatment modalities for many refractory diseases.

**Figure 5:**
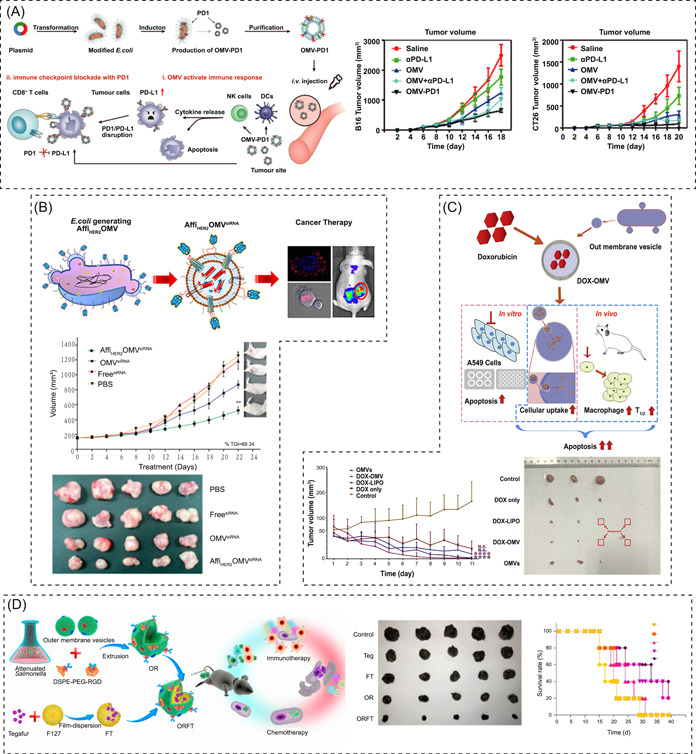
BEVs‐based therapeutic application in cancer treatment. (A) schematic illustration of the development of genetically engineered BEVs that modified with PD1 overexpression, leading to enhanced immune activation and subsequent increased antitumor efficacy. Reproduced with permission [257]. Copyright 2020, American Chemical Society. (B) Schematic representation of the BEVs‐based small interfering RNA delivery system, by which si‐HER2 are translocated to tumor cells and contribute to targeted gene silencing and significant tumor growth regression. Reproduced with permission [251]. Copyright 2014, American Chemical Society. (C) schematic illustration of BEVs‐based drug delivery system, doxorubicin‐loaded BEVs exerted improved efficacy of tumor chemoimmunotherapy. Reproduced with permission [255]. Copyright 2020, Elsevier. (D) schematic illustration of combination of BEVs and nanomedicine approaches, BEVs coated on drug‐loaded polymeric micelles were highly effective cancer immunotherapy and chemotherapy. Reproduced with permission [256]. Copyright 2020, American Chemical Society. BEV, bacterial extracellular vesicle.

BEVs can also serve as potent drug delivery vehicles based on their intrinsic properties, such as loading capacity, specific targeting, and temperature stability. BEV‐based delivery platforms present distinct advantages compared with conventional nanomaterials, such as liposomes, polymers, and metal‐based nanoparticles, especially for intercell communication [241]. Researchers have developed various strategies to ensure successful loading of bioactive substances into BEVs; compounds or biomolecules of interest can be either actively encapsulated by parent cell engineering approaches or by directly modifying BEVs using exogenous incubation, permeabilization, electroporation, among others [242–245]. BEVs can be loaded with various effective proteins, RNA, and small‐molecule drugs, thereby preventing enzymatic degradation, enabling controlled release, achieving distant delivery, and synergistically enhancing therapeutic effects [246–249]. In a dextran sodium sulfate‐induced colitis animal model, *Bacteroides thetaiotaomicron* BEVs loaded with the human therapeutic protein keratinocyte growth factor‐2 (KGF‐2) in a stable form, are found to alleviate disease severity and promote intestinal epithelial repair and recovery [250]. In particular, the specificity of engineering BEVs to target tumor cells by conjugating them with ligands for tumor cell receptors, and their ability to protect their contents, such as chemotherapeutic agents, from protease or nuclease degradation may confer BEV‐based anticancer therapies with greater specificity and fewer adverse side effects compared to traditional indiscriminate chemotherapies [242]. A previous study developed engineered *E. coli* BEVs decorated with HER2 ligands and loaded with specific antitumor siRNA, which showed high specificity to cancer cells and impressive tumor cell‐killing effects [251]. Similarly, epidermal growth factor (EGF) receptors are commonly expressed on the surface of numerous tumor cells of epithelial origin; thus, engineered BEVs expressing EGF on the surface show high specificity to tumor cells and significantly hinder tumor growth and reduce tumor burden without notable adverse effects when loaded with chemotherapeutics [252]. Furthermore, BEV‐based anticancer therapies may also be combined with other novel techniques, such as immunotherapeutics and nanotechnologies, to amplify the therapeutic efficacy [253–257] (Figure 5A–D). To further enhance the functionality and expandability of BEVs, researchers have designed and constructed a hybrid eukaryotic–prokaryotic vesicle (EPV) nanoplatform by fusing attenuated *Salmonella* BEVs with tumor cell membranes and vesicles, which evoke potent tumor‐specific immune responses. In addition, the EPV platform possesses high expandability to load the complementary photothermal modality and protects mice from melanoma challenge [258]. Thus, the combination of BEVs and other conventional treatments may shed light on novel therapeutics for cancer.

## CONCLUDING REMARKS

Over the past decades, potent associations between microbiota homeostasis and human health, especially gut microbiota, have been intensively investigated. BEVs derived from various commensal and pathogenic bacteria have been shown to profoundly participate in a wide range of physiological and pathological processes and show great potential in biomedical applications. Within this context, our knowledge regarding the origin, biogenesis, composition, biofunctions, and clinical applications are rapidly expanding. Accumulating evidence has demonstrated that BEVs generation and release present a conserved and active secretory mechanism in which molecules of definite composition are destined to be entrapped in BEVs, thereby conferring their versatile biofunctions, including biofilm formation, antibiotic resistance, virulence factor delivery, inflammation, and immunomodulation. The biological properties of BEVs, including their size, content, and yield, are significantly influenced by multiple genetic and environmental factors. Moreover, BEVs shuttled bioactive molecules throughout the body may provide a novel observation window for the pathogenesis of diverse systemic diseases. Furthermore, owing to their distinct characteristics of high biocompatibility, low toxicity, immunogenicity, ease of manipulation, and potential for cell targeting, BEVs are of great interest for broad utilization in clinical settings, including disease diagnoses, vaccine formulation, and novel therapeutics.

Despite the recent remarkable achievements in our knowledge of BEVs' generation and preliminary application, several puzzles remain regarding the underlying mechanism of BEVs' biogenesis and their exact functional role in disease. For example, BEVs can be released by cells in distinct forms, depending on their growth conditions, extent of stress, and species. Is there a universal pathway through which cells sense stimuli to drive BEV generation? How are cargo sorted and selectively packaged in BEVs? How do external factors influence the BEVs' content and release? How do BEVs distribute throughout the body and interact with host cells? Are they destined for specific target cells? Are BEVs' contents that have not been identified endowed with specific biological functions? In the future, deeper and broader cooperation in areas of high‐throughput sequencing, multiomics approaches, artificial intelligence analytics, and nanobiotechnology may provide new lights on resolving these questions and facilitating translational applications. In addition, there are several ongoing challenges and technical issues in the biomedical utilization of BEVs. For example, techniques for BEV preparation, purification, and identification are still in their infancy, and more reliable, reproducible, efficient, and standardized BEV production procedures are needed. BEV‐associated immunogens, such as LPS, are critical for vaccine development but can also elicit immunotoxicity and raise safety concerns. Thus, balancing the high efficacy of immunogenicity and eliminating immunotoxicity will be an intriguing topic in the future. Furthermore, BEV‐based cell‐ or tissue‐targeted therapeutics have shown great promise in precision medicine, especially for cancer treatment, while their accuracy, specificity, and efficiency need to be further improved.

Collectively, research in the field of BEVs is rapidly evolving and will continue to revolutionize our understanding of BEVs' biology, particularly BEVs' biogenesis, biofunctions, and biomedical applications. The BEV‐based platform is expected to offer an innovative way to think about pathogenesis and treatment modalities for various conditions (Table 1).

**Table 1:** Characteristics of the main types of extracellular vesicles (EVs).

	Type	Size (nm)	Biogenesis	Reference
Eukaryotic EVs	Exosomes	30–150	Released by multivesicular bodies fusing with plasma membrane	[30, 31, 71]
	Microvesicles	100–1000	Outward budding of the plasma membrane	[30, 31, 71]
	Apoptotic bodies	>1000	Programmed cell death	[30, 31, 71]
Bacterial EVs	Outer membrane vesicles	20–300	Blebbing of the outer membrane of Gram‐negative bacteria	[39, 71, 110]
	Outer‐inner membrane vesicle	60–160	Explosive cell lysis and cell budding of Gram‐negative bacteria	[39, 43, 44]
	Cytoplasmic membrane vesicles	20–400	Extrusion of the cell membrane and release through cell wall pores or holes of Gram‐positive bacteria	[39, 71, 110]

## AUTHOR CONTRIBUTIONS

**Rongjin Luo**, **Yanmin Chang**, and **Huaizhen Liang**: conceptualization, literature review, and writing ‐ original draft; **Weifeng Zhang** and **Yu Song**: literature review, visualization; **Gaocai Li** and **Cao Yang**: supervision, writing ‐ review & editing, and funding acquisition. All authors have read the final manuscript and approved it for publication.

## CONFLICT OF INTEREST STATEMENT

The authors declare no conflicts of interest.

## Data Availability

No new data and script were used in this paper. Supporting Information materials (figures, tables, scripts, graphical abstract, slides, videos, Chinese translated version and updated materials) may be found in the online DOI or iMeta Science http://www.imeta.science/.
